# The Role of Lipids in the Initiation of α-Synuclein Misfolding

**DOI:** 10.3389/fcell.2020.562241

**Published:** 2020-09-15

**Authors:** Martin Kiechle, Veselin Grozdanov, Karin M. Danzer

**Affiliations:** Department of Neurology, Ulm University, Ulm, Germany

**Keywords:** alpha synuclein (α-syn), alpha synuclein accumulation, Parkisnon’s disease, lipid turnover, alpha synuclein oligomers

## Abstract

The aggregation of α-synuclein (α-syn) is inseparably connected to Parkinson’s disease (PD). It is now well-established that certain forms of α-syn aggregates, oligomers and fibrils, can exert neurotoxicity in synucleinopathies. With the exception of rare familial forms, the vast majority of PD cases are idiopathic. Understanding the earliest molecular mechanisms that cause initial α-syn misfolding could help to explain why PD affects only some individuals and others not. Factors that chaperone the transition of α-syn’s physiological to pathological function are of particular interest, since they offer opportunities for intervention. The relationship between α-syn and lipids represents one of those factors. Membrane interaction is crucial for normal cellular function, but lipids also induce the aggregation of α-syn, causing cell toxicity. Also, disease-causing or risk-factor mutations in genes related to lipid metabolism like PLA2G6, SCARB2 or GBA1 highlight the close connection between PD and lipids. Despite the clear link, the ambivalent interaction has not been studied sufficiently so far. In this review, we address how α-syn interacts with lipids and how they can act as key factor for orchestrating toxic conversion of α-syn. Furthermore, we will discuss a scenario in which initial α-syn aggregation is determined by shifts in lipid/α-syn ratio as well as by dyshomeostasis of membrane bound/unbound state of α-syn.

## Introduction

The abnormal aggregation of α-synuclein (α-syn) in the central nervous system defines neurodegenerative diseases such as Parkinson’s disease (PD), Dementia with Lewy bodies (DLB) and Multiple system atrophy (MSA) (Spillantini and Goedert, 2000). They all share common neuropathological hallmarks as a result of α-syn accumulation, known as Lewy bodies (LBs) and Lewy neurites (LNs). Although these age-related diseases are predominantly idiopathic, genetic studies demonstrate that the protein α-syn can directly contribute to pathologic events (Stefanis, 2012). However, initial mechanisms of α-syn misfolding that precede LB and LN formation remain elusive. This review summarizes recent advances in understanding the causes of α-syn aggregation.

α-Syn is an abundant protein in brain cells and is found alongside its nuclear localization mainly at the synapse of neurons (Iwai et al., 1995; Kahle et al., 2000; Pinho et al., 2018). About 3,000 α-syn molecules are present within a single synaptic bouton of a cortical rat neuron, highlighting its enormous concentration at synaptic terminals (Wilhelm et al., 2014). Since α-syn localizes to the synaptic compartment at a very late stage of synapse development, it was thought early on that it functions as a modulator of synaptic plasticity rather than taking part in synaptogenesis (Withers et al., 1997). Currently, we are in the process of deciphering the physiological functions of α-syn. Although there is no universal consensus, its function has increasingly been associated with regulating synaptic vesicle exo- and endocytosis (reviewed recently in Sulzer and Edwards, 2019). However, it was only in the last decade that we learned that α-syn can adopt a multimeric state at the presynapse, e.g., for promoting the SNARE complex formation or clustering synaptic vesicles and thereby attenuating neurotransmitter release (Burre et al., 2014; Wang et al., 2014). Studies showing that monomeric α-syn is the predominant species in the cytoplasm of cells were often carried out in (neuronal) cell lines that do not form a synaptic compartment (Theillet et al., 2016). However, (synaptic) membrane binding may be crucial for the temporal higher-order multimeric conformation and phospholipids seem to bear chaperone-like characteristics in this process. α-Syn is thereby in a dynamic conformational transition between α-helically structured in the membrane-bound situation – and monomeric, natively unfolded in the unbound state (Rovere et al., 2018). The classic paradigm, that α-syn is a natively unfolded protein has therefore been broadened by various findings in recent years (Cole et al., 2002; Bartels et al., 2011; Dettmer et al., 2013; Gould et al., 2014). The behavior of α-syn at the synapse is distinct from other proteins that also associate with the vesicle surface, such as synapsin I. In contrast to synapsin I, α-syn is highly mobile and binds synaptic vesicles only transiently as shown by fluorescence recovery after photobleaching (FRAP) experiments using GFP-tagged α-syn (Fortin et al., 2005). Remarkably, the localization of α-syn to the bouton was thereby dependent on neural activity, and α-syn quickly dispersed after strong stimulation.

## The Structure of α-Synuclein Determines Its Lipid Binding Characteristics

The observation that α-syn is not tightly associated with synaptic vesicles might explain why it is usually purified as (monomeric) cytosolic protein from brain extracts, and not as constituent of the synaptic vesicle fraction (Takamori et al., 2006). However, isolation of synaptosomes under physiological salt conditions and immunoelectron microscopy verified the vesicle-binding properties of α-syn and showed that the majority (74.2%) of immunogold-labeled α-syn molecules were found on the surface of synaptic vesicles throughout the terminals (Vargas et al., 2017). But how is this transient affinity of α-syn to lipid membranes accomplished? The answer to this may lie in the structure of α-syn. Firstly, the primary structure of the 140 amino acids-long protein α-syn can be divided in three distinct regions. The N-terminal region (aa 1–60) contains imperfect repeats of 11 amino acids with a KTKEGV consensus sequence, predicted to form an amphipathic α-helix that resembles those found in apolipoproteins and known to bind and penetrate membranes (Clayton and George, 1998; Ahn et al., 2006). Secondly, the central domain (aa 61–95), also known as NAC domain (non-Aβ component of AD amyloid), is hydrophobic and prone to aggregation due to its propensity to form β-sheet-rich oligomeric conformations (Ueda et al., 1993; Giasson et al., 2001; Stefanis, 2012). This region further contains elements of the KTKEGV consensus sequence and together with the N-terminal domain, approximately two-thirds of the whole protein can form α-helices upon lipid binding. Thirdly, the negatively charged C-terminal domain (aa 96–140) is characterized by 33% acidic amino acids (Asp/Glu) and a proline-rich region that is responsible for the disordered C-terminal structure. However, not so long ago it was also suggested that the C-terminus associates with membranes in the presence of calcium (Lautenschlager et al., 2018). These intrinsic preconditions of the α-syn sequence as well as the lipid composition of the membrane to be bound including hydrophobicity, charge, or membrane curvature, most likely modulate membrane binding of α-syn. By mimicking synaptic-like lipid membranes in a solid-state and solution NMR spectroscopy approach, it was found that three different domains within the α-syn sequence interact differently with lipid membranes (Fusco et al., 2014). The most N-terminal α-helix (aa 6-25) anchored α-syn to the membrane surface with high affinity upon first contact, without being particular about lipid composition. In contrast, a central domain (aa 26-97) bound membranes with different intensities depending on lipid composition, suggesting a potential role as “modulating membrane sensor.” The C-terminal region (98-140) interacted only weakly with the membrane and behaved highly dynamic and independent from the other domains. It is therefore suggested that the central domain (aa 26-97) dictates whether to bind, or not to bind the lipid. Intriguingly, all familial mutations causing early- or late-onset PD (A30P, E46K, H50Q, G51D, A53T, A53E) are located within this crucial sequence, which could affect the specificity of membrane affinity or influence the N-terminal anchor. It was indeed found that some pathogenic α-syn mutants reduce lipid binding behavior, of which G51D and especially the helix-breaker mutation A30P had the strongest effect (Ruf et al., 2019). The A30P mutation was further found to negatively influence the N-terminal anchor, reasoning the substantial reduction in binding affinity of α-syn to small unilamellar vesicles (Fusco et al., 2016). However, for the early onset PD variant E46K, exactly the opposite was observed. The N-terminal anchor region (aa 6-25) was extended to aa 42, thereby increasing the membrane affinity.

## Lipid Binding Modulates Initial α-Synuclein Aggregation

**FIGURE 1:**
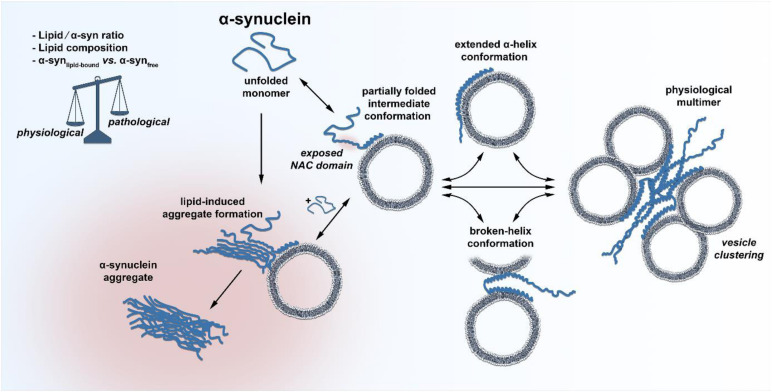
Schematic illustration of the discussed conformational states of α-syn. The unfolded, unbound and monomeric α-syn adopts a partially folded intermediate conformation at its very N-terminus upon membrane binding, leaving the NAC domain exposed for potential primary nucleation. Freely available monomeric α-syn could bind to the hypothesized intermediate conformation, facilitating initial lipid-induced amyloid oligomer and fibril formation. Under physiological conditions, α-syn quickly adopts an extended α-helical or a broken-helix conformation, which can also form physiological multimers that cluster synaptic vesicles. A change in the lipid/α-syn ratio, the lipid composition or the fraction of membrane bound vs. unbound α-syn could shift the balance between physiological and pathological paths.

Does decreased or enhanced membrane affinity, based on amino acid exchange influence the propensity of α-syn to form abnormal aggregates? First of all, the aggregation kinetics of α-syn depend on a range of different solution conditions, of which the pH has the strongest impact (Buell et al., 2014). Aggregation kinetic experiments carried out at pH 6.5 in the presence of negatively charged lipid vesicles demonstrated that the rate of lipid-induced aggregation is indeed different for disease-associated α-syn mutations (Flagmeier et al., 2016). In this set of experiments, only the A53T mutation showed a clear enhancement for initial aggregate formation, whereas A30P was only slightly increased and E46K was much slower compared to wild type. In addition, the process of fibril elongation was only weakly affected by the mutants. Most importantly, Galvagnion et al. suggested that under quiescent conditions, the lipid-to-α-syn ratio is the most determinant factor for α-syn misfolding (Galvagnion et al., 2015). At high ratios of lipid/α-syn, only very little free, monomeric α-syn was present since the large excess of lipid resulted in all α-syn molecules being bound α-helically to the surface of membranes. In this experimental condition, α-syn aggregation was below the detection limit. A decrease in lipid concentration (low lipid/α-syn ratio), however, caused that α-syn was only partially bound to membranes and that a substantial proportion was freely available as a monomer in solution. This condition greatly favored the essential primary nucleation step on the lipid surface that gives rise to fibril formation. By using atomic force microscopy, Galvagnion et al. further found that only a fraction of vesicle-bound α-syn was able to serve as active nucleation seed, from which fibrils could sprout. Through these experiments, one could set up a hypothesis that in the situation where α-syn is bound to the membrane or is in the process of forming the α-helical structure, an aggregation-susceptible intermediate conformation could occur, which freely, unfolded monomeric α-syn could bind if available, resulting in abnormal aggregation (Figure 1). Since natively unfolded proteins hold heterogeneous conformational states per se and the free energy landscape of α-syn is highly dynamic, the existence of such an intermediate conformation is likely (Allison et al., 2009). Indeed, the presence of a partially folded intermediate conformation of α-syn has been suggested many years back (Uversky et al., 2001). A decrease in pH or increase in temperature resulted in a partially folded intermediate conformation of α-syn. The concentration of lipids could therefore represent an additional intracellular factor that shifts the equilibrium of α-syn between an aggregation-prone intermediate conformation and its natively folded/unfolded state. Future cryo-electron microscopy experiments could provide insights into such a state and deliver a complete view of membrane-induced α-syn folding. The lipid-water interface would be vital for primary nucleation, since it is known that interfaces play important roles in the aggregation of α-syn (Campioni et al., 2014). This simplified scenario for initial aggregate formation determined by a low lipid/α-syn ratio as well as altered concentrations of α-syn_lipid–bound_ and α-syn_free_ could explain several PD-associated findings, like the α-syn-dosage effect for the development of PD. Increased α-syn expression levels by genetic locus duplication/triplication, variabilities in SNCA-promoter region REP1 or somatic copy number gains of α-syn all demonstrate the close relation of α-syn concentration and PD age of onset (Singleton et al., 2003; Chartier-Harlin et al., 2004; Maraganore et al., 2006; Fuchs et al., 2008; Mokretar et al., 2018). The lipid/α-syn ratio is likely to be lowered by elevated levels of α-syn at the synapse, thereby increasing the chance for lipid-induced spontaneous aggregation events. Another factor responsible for a shift in the lipid/α-syn ratio could be aging, which remains the highest risk factor for the development and progression of PD (de Lau and Breteler, 2006; Collier et al., 2011). More than half of the human brain’s dry weight is made up of lipids, and the incredible heterogeneous lipid composition of the brain changes significantly with age (Naudi et al., 2015). Depending on the class of lipids and the area of the brain, the concentration of some lipids increases with age, but the majority is substantially decreased. In particular, levels of cholesterol, gangliosides or glycerophospholipids, all enriched at synapses, are reduced with age (Rappley et al., 2009; Ledesma et al., 2012). Age-related lipid alterations in the brain could therefore lower the local lipid/α-syn ratio and increase the primary nucleation rate. It is worth mentioning that α-syn aggregation is not only triggered by phospholipids with small acidic headgroups, but also by anionic lipids that contain larger oligosaccharide headgroups like gangliosides (Gaspar et al., 2018). It was found that all major brain gangliosides were significantly decreased in male PD patients compared to healthy controls (Seyfried et al., 2018). Pilot studies further showed that treating PD Patients with GM1 ganglioside could provide a disease modifying therapy and that GM1 administration reduced the size of α-syn aggregates in a rodent AAV-α-syn overexpression model (Schneider et al., 2013, 2015, 2019). However, research of human brain lipidomics of different brain areas or cell types during aging in health and disease is limited. Prospective studies could provide exciting insights into whether lipid alterations (at the synapse) in the aged-brain modify the behavior of α-syn.

Aberrant interaction of α-syn with biological membranes like enhanced membrane-binding also fits in the concept of lipid/α-syn ratio induced aggregation. As previously mentioned, the E46K mutation has a greatly increased membrane affinity. This mutation disrupts an N-terminal KTKEGV motif to KTKKGV and drastically destabilizes physiological aggregation-resistant multimers of α-syn, leading to enhanced levels of aggregation-prone monomeric α-syn (Dettmer et al., 2015). In addition, a recent report argues for a loss of α-syn’s membrane curvature-sensing ability due to E46K mutation, thereby misdirecting α-syn to non-physiological lipid interactants (Rovere et al., 2019). By introducing two additional E > K mutations in other KTKEGV motifs (E35K and E61K, termed “3K” with the E46K mutation), this effect was dose-dependently amplified, resulting in increased monomeric conformations of α-syn and induction of neurotoxic inclusions. By increasing monomeric α-syn, which additionally shows an increased spatio-temporal membrane binding behavior, the probability of primary nucleation on the surface of lipids would be greatly increased. Generation of a 3K α-syn mouse model strengthened the *in vitro* findings and impressively mirrored key characteristics of PD (Nuber et al., 2018). Due to increased membrane affinity and potentially an increase in the partially folded intermediate state, 3K α-syn accumulated extensively at presynaptic vesicles and formed Proteinase-K resistant, phosphorylated, and truncated α-syn deposits. Even young 3K mice showed large neuronal lipofuscin-like autofluorescent, but α-syn-positive deposits that increased with age and evolved into huge spherical aggregates with filamentous structures and a lipofuscin-rich center. This finding in particular is interesting in the light of the recent report, that LBs in the brain of PD patients consisted largely of fragmented lipids, vesicles and organelles (Shahmoradian et al., 2019). The combination of enhanced membrane association, the inability to form physiological multimers, and the low lipid/α-syn ratio by α-syn overexpression could reason the striking phenotype of the 3K model. But how do the familial α-syn mutations A30P and G51D fit into the picture? To start with, the reduced lipid binding properties of A30P and G51D do not mean that lipid binding is completely abolished, but rather that the affinity is reduced compared to wildtype α-syn. Since they also destabilize physiological multimers and thereby increase the moiety of aggregation-prone monomeric α-syn, in theory only the rate of primary nucleation would be slower. Indeed, A30P as well as G51D were found to attenuate aggregation, although inconsistent reports for A30P exist (Lemkau et al., 2012; Fares et al., 2014). In addition, elevated levels of a specific conformation of the N-terminal amphipathic α-helix termed SL1 has been observed for A30P, E46K and A53T compared to wildtype (Bodner et al., 2010). Here, only a short α-helix comprising residues 3–25 was membrane-bound, leaving the hydrophobic NAC region dynamically disordered and prone for disease-associated aggregate formation. However, an alternative pathologic loss of function mechanism for A30P and G51D, independent of lipid-induced aggregation, cannot be excluded.

## The Lipid Composition Alters α-Synuclein Aggregation

Membrane-interaction is part of the physiological function of α-syn. The lipid-binding properties are predefined by the first 60 amino acids of the N-terminal sequence with its 18% positively charged lysine residues, directing the affinity for anionic lipids. Upon membrane binding, α-syn forms amphipathic α-helices. Several conformations have been observed, like a single extended α-helix that is over 90 aa long, the previously mentioned short α-helix comprising residues 3–25 or two α-helices interrupted by a short break (Chandra et al., 2003; Jao et al., 2004, 2008; Bodner et al., 2010). Moreover, α-syn was found to be a membrane curvature-sensing protein, preferentially binding to small unilamellar vesicles with diameters similar to those of synaptic vesicles (Middleton and Rhoades, 2010). The mechanisms by which lipids orchestrate the physiological multimerization or induce pathological aggregation of α-syn, however, have yet to be clarified. Several studies analyzed physical and chemical properties of biological membranes or lipids and how they influence the aggregation propensity of α-syn. Amyloid fibril formation of α-syn has been observed for lipids with high solubility in aqueous solution and short hydrocarbon chains (Galvagnion et al., 2016). It was further discovered that exosomes with their high GM1 and GM3 ganglioside concentration provide an environment for accelerated α-syn aggregation, an interesting finding considering exosomal cell-to-cell transmission of α-syn oligomers (Danzer et al., 2012; Grey et al., 2015). Likewise, oxidized cholesterol metabolites, that were found to be increased in brains of patients with LBD, induced fibrillation of α-syn (Bosco et al., 2006). *In vitro* experiments also showed that polyunsaturated fatty acids (like α-linolenic acid or eicosapentaenoic acid) alone or esterified with phospholipids promote the formation of α-syn oligomers along with cytotoxicity, whereas saturated fatty acids (like stearic acid or arachidic acid) decreased levels of α-syn oligomers (Perrin et al., 2001; Sharon et al., 2003; Snead and Eliezer, 2014). These data, showing that lipids of different classes are able to induce disease-associated misfolding of α-syn, favor a model in which lipids play a significant role for the development of synucleinopathies, although the context of a relevant biological setting was mostly missing. Overexpression of α-syn in several PD-related cell culture systems and *in vivo* led to higher levels of di-/triglycerides with increased concentrations of unsaturated fatty acids, especially oleic acid (Fanning et al., 2019). This change in lipid class composition was accompanied by buildup of aggregated and phosphorylated α-syn species along increased cytotoxicity. The enrichment of oleic acid-containing lipids in membranes most likely enhances membrane fluidity and affects its curvature. The authors suggested that the rate of membrane-associated α-syn is enhanced by increased oleic acid levels, which mediated α-syn toxicity. Although the origin of higher amounts of oleic acid is unknown, decreasing the levels by inhibiting its rate-limiting enzyme stearoyl-CoA desaturase (SCD) proved to be protective and broke the vicious pathological circle (Fanning et al., 2019). Targeting SCD is a promising therapeutic strategy, which was also found independently by Vincent et al. Here, inhibiting SCD reduced α-syn toxicity in human induced pluripotent stem cell neuronal models (Vincent et al., 2018). These studies were the first to target α-syn oligomerization by exploiting its membrane-binding nature.

## Relation Between Lipids, Different Oligomeric α-Synuclein Species and Toxicity

From what it described above, it is clear that lipids can act as trans-factors and modulate α-syn aggregation. However, different trans-factors favor the formation of different oligomeric species with distinct physico-chemical and toxic characteristics (Bousset et al., 2013). It is therefore only logical to ask if lipid binding favors the formation of specific oligomeric species with distinct toxicity. To date, this question remains to be answered. Suzuki et al. employed a GBA1 knockout model to demonstrate that disbalance of glycosylceramide results in the accumulation of PKA-resistant α-syn (Suzuki et al., 2015). However, the exact species of α-syn remained unspecified. The spectrum of different oligomeric α-syn species is rapidly growing and populated by a very high number of small to intermediate molecular-weight α-syn oligomers and different strains of high molecular-weight fibrils. Virtually all of the characterized species have been ascribed some toxic function (for an exhaustive review, see Ingelsson, 2016; Alam et al., 2019). Oligomers can induce cytoskeletal perturbances, ER stress, mitochondrial dysfunction, increased ROS production, ion flux dysbalance, synaptotoxicity and inhibition of the cellular protein synthesis and degradation (Lindersson et al., 2004; Danzer et al., 2007, 2009; Vekrellis et al., 2011; Colla et al., 2012; Choi et al., 2013; Deas et al., 2016). Lipid binding itself is required for some of these observed toxic effects: small, β-sheet-rich oligomers form pores in lipid bilayer membranes and result in detrimental ion flux and vesicle rupture (Danzer et al., 2007; Flavin et al., 2017). Furthermore, α-syn oligomers and fibrils have differential, detrimental effects on anterograde axonal transport (Prots et al., 2013). Large, fibrillar aggregates of α-syn show less direct toxicity to cells; however, they are unique in their capability to propagate α-syn aggregation by recruiting endogenous α-syn and result in complex synucleinopathies in animal models (Alam et al., 2019). Importantly, the toxic effects of oligomeric α-syn species are observed not only *in vitro*, but also *in vivo* (Winner et al., 2011). The traditional view on α-syn oligomers suggests the physiological existence of soluble, endogenous non-toxic α-syn oligomers that are converted to toxic species by pathologic mechanisms. These “physiological” oligomers were recently joined by the stable, membrane-bound tetramers described above. In this view, the (pathologic) binding of α-syn to lipids may stabilize aggregation-prone conformations and thus favor or inhibit the formation of toxic soluble oligomers. However, Killinger and colleagues recently challenged this traditional view by suggesting an alternative, monomer-only and lipid-centric hypothesis postulating that the apparent, soluble α-syn oligomers are in fact conformations of membrane-bound α-syn (Killinger et al., 2019). In this model, the formation of any α-syn oligomers is detrimental and the critical pathological step is the conversion of monomers into oligomers. The distinction between these two models remains technically challenging, but it will have tremendous impact on the choice of appropriate therapeutic strategy.

## The Synapse as Early Point of Departure From Physiological to Pathological

Different anatomical sites and cell types have been proposed to be the site of initial α-syn aggregation. Indeed, α-syn is expressed at relatively high levels even in peripheral tissues. Erythrocytes, immune cells and fibroblasts are several cell types that can contain high levels of endogenous (and probably oligomeric) α-syn (Nakai et al., 2007; Hoepken et al., 2008). Interestingly, most of the observations of α-syn in peripheral cell types include processes with increased membrane dynamics. For example, α-syn expression and localization at cellular membranes is increased during the enucleation of erythrocyte precursors and during the phagocytosis of extracellular material (Nakai et al., 2007; Gardai et al., 2013; Abd-Elhadi et al., 2015). However, several factors may render CNS neurons specifically vulnerable to initial α-syn aggregation: different abundance of β-synuclein and molecular chaperones, increased metabolic rate, increased melanin and ROS, high turnover of membranes. Since α-syn localizes largely to the synapse of neurons, it is worth thinking about which synaptic membranes contain large amounts of di- or triglycerides. Lipidome analysis found porosomes, a domain of the presynaptic synaptosome, to be enriched in diglycerides, and that synaptic vesicles have high concentrations of triglycerides and sphingomyelins compared to the surrounding synaptosomal membrane (Lewis et al., 2014, 2017). Moreover, it is known for a long time that sphingomyelins are enriched in Lewy bodies, and that the activity of enzymes related to sphingolipid metabolism in the brain changes with age (den Jager, 1969; Sacket et al., 2009). An emerging consensus from various laboratories suggest the synapse as starting point for pathologic events in PD with axon terminals representing the initial site for α-syn aggregate formation. From a genetic perspective, several genes implicated in PD pathogenesis code for proteins related to synapse function (Soukup et al., 2018). First α-syn-related dysfunctions in rodent models were detected at the synapse, such as altered synaptic vesicle pool, deficits in neurotransmitter release or even redistribution of SNARE proteins (Garcia-Reitbock et al., 2010; Nemani et al., 2010; Scott et al., 2010; Phan et al., 2017). A substantial loss of synaptic terminals that preceded the death of dopaminergic neurons was observed in a mouse model with overexpression of mutant α-syn in the background of elevated dopamine (Mor et al., 2017). This state is reminiscent of human post-mortem brain studies that suggested synaptic decay as earliest pathology in PD (Beach et al., 2008; Cheng et al., 2010). In DLB patients, synaptic terminals were suggested to be the loci for α-syn aggregation and axonal retrograde transport with LB formation is the cellular counteracting strategy (Kramer and Schulz-Schaeffer, 2007). Using protein-fragment complementation assays *in vivo*, we have recently provided additional evidence that α-syn oligomerizes at the presynapse, giving rise to pathologically relevant α-syn species (Kiechle et al., 2019). Understanding how lipids are involved in synaptic α-syn aggregation during the aging process could hold promising treatment opportunities. The possibility that lipids act as effectors causing distinct α-syn strain variants is also currently being contemplated in the field of synucleinopathies (Ikenaka et al., 2019). Hence, modulating neuronal lipid synthesis in the brain could protect the synapse as most vulnerable compartment of neurons and allows to explore new therapeutic avenues for PD, DLB and MSA.

## Author Contributions

MK designed and wrote the manuscript. VG and KD wrote and edited the manuscript. All authors contributed to the article and approved the submitted version.

## Conflict of Interest

The authors declare that the research was conducted in the absence of any commercial or financial relationships that could be construed as a potential conflict of interest.
